# Supplementary material for: A critical role of AREG for bleomycin-induced skin fibrosis
Source: Cell Biosci. 2021 Feb 23;11:40. doi: 10.1186/s13578-021-00553-0 (PMC7903615; doi:10.1186/s13578-021-00553-0)
Supplement: Supplementary file 3 — Additional file 3. Sirius red/fast green staining of skin tissues. [file 13578_2021_553_MOESM3_ESM.pdf]

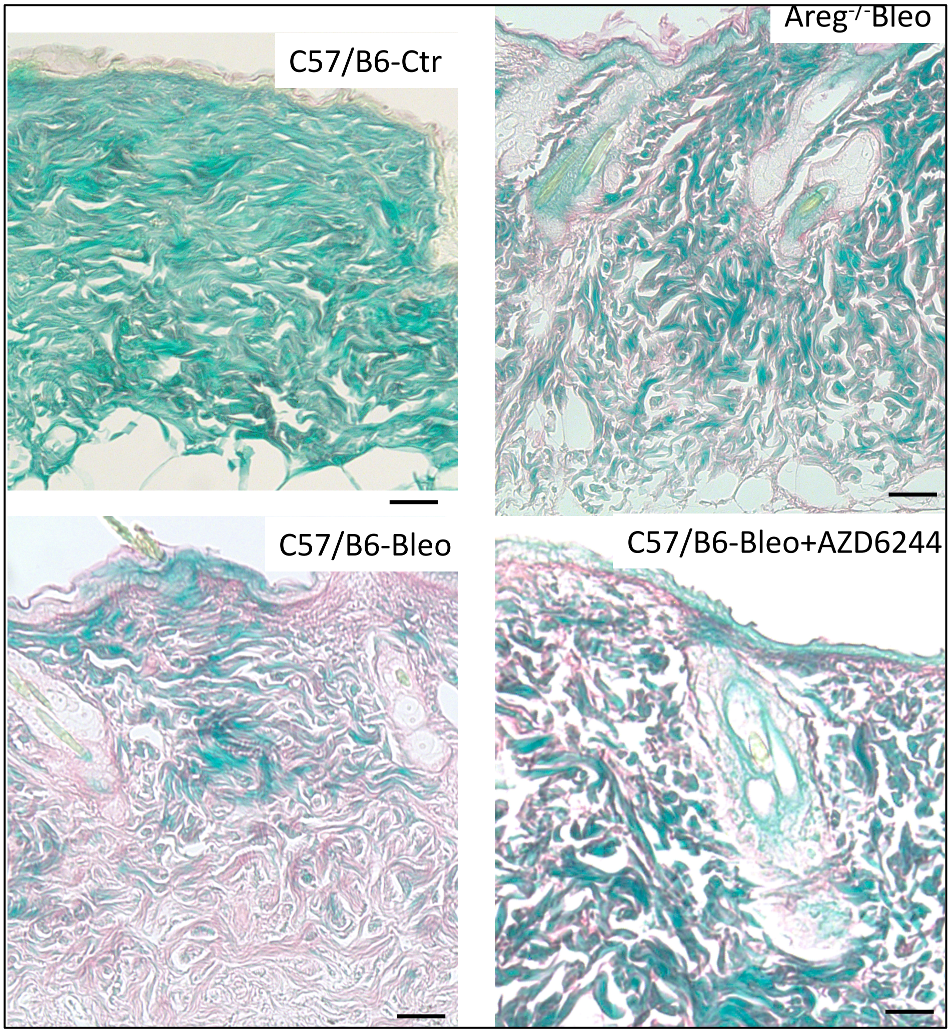

Supplementary data 3- Sirius red/Fast green staining of mouse skin tissues. Skin samples were fixed in formalin and embedded in paraffin. Five-micrometer tissue sections were stained first with 0.01% Fast green for 15 minutes, and then with 0.1% Sirius red and 0.04% Fast green dissolved in water saturated with picric acid for 5 minutes (see Methods for details). Collagens were stained in red by Sirius red, and non-collagen proteins were stained in green by fast green. Please note that bleomycin-treated skin tissues (indicated as Bleo) have more Sirius red staining (red) in the wild type C57/B6 mice whereas skin tissues with Areg knockout (shown as Areg$^{-/-}$) or MEK inhibitor AZD6244 treatment (shown as AZD6244) showed less Sirius red staining. Bar indicates 200μm
