# Supplementary material for: A critical role of AREG for bleomycin-induced skin fibrosis
Source: Cell Biosci. 2021 Feb 23;11:40. doi: 10.1186/s13578-021-00553-0 (PMC7903615; doi:10.1186/s13578-021-00553-0)
Supplement: Supplementary file 4 — Additional file 4. A summary of single cell RNAseq analyses. [file 13578_2021_553_MOESM4_ESM.pdf]

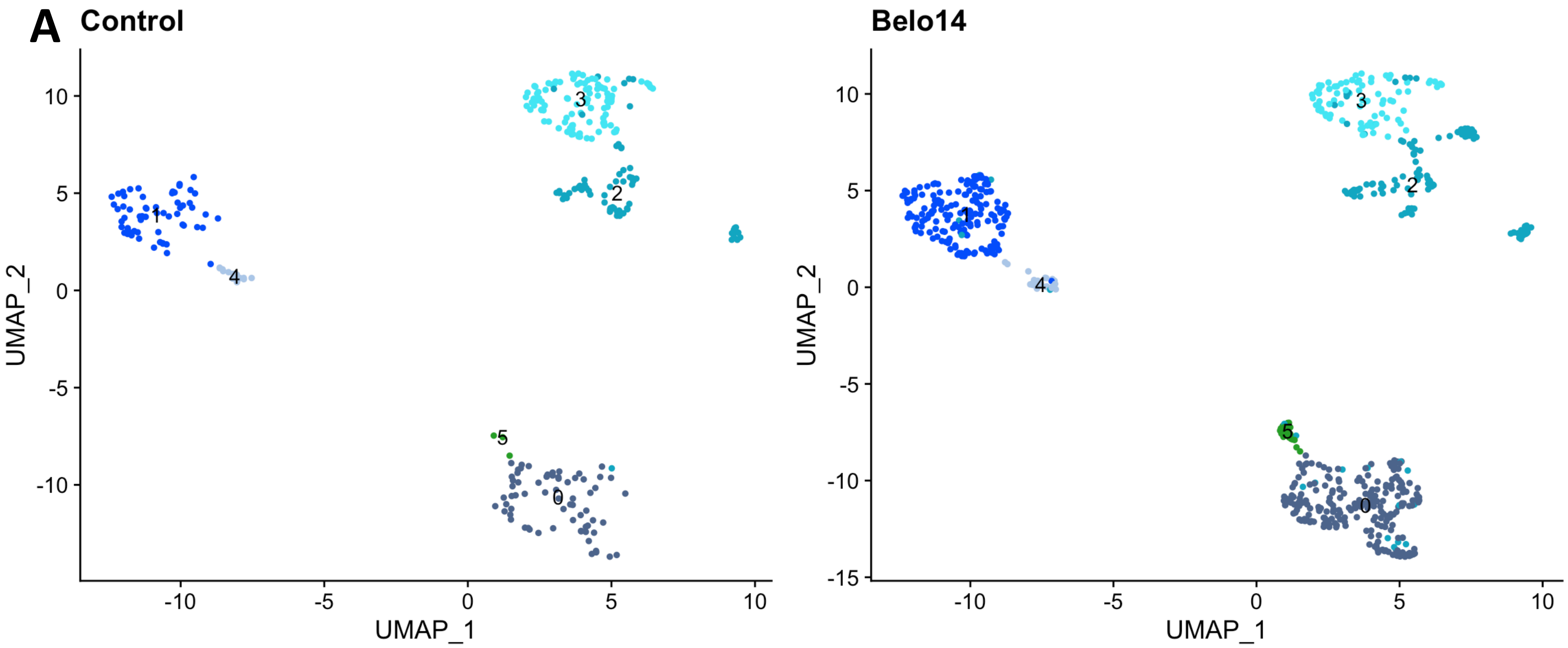

B

Areg expression in clusters	Cluster 0	1	2	3	4	5
Control	-	+	-	-	-	-
Bleomycin-injected (Bleo14)	-	-	+	+	-	-

Supplementary data 4- A summary of single cell RNAseq analyses.

A shows distribution of cell clusters in control and bleomycin-treated mouse skin.

B summarizes *Areg* expression in different cell clusters. Cluster 0 contains macrophages and monocytes. Cluster 1 contains keratinocytes. Cluster 2 contains immune cells and endothelial cells. Cluster 3 contains fibroblasts (and myofibroblasts). Cluster 4 contains NK cells and T cells. Cluster 5 contains dendritic cells. Areg is expressed in cluster 1 in the control skin and in clusters 2 and 3 in bleomycin-treated skin tissues.
